# H-NS Nucleoid Protein Controls Virulence Features of *Klebsiella pneumoniae* by Regulating the Expression of Type 3 Pili and the Capsule Polysaccharide

**DOI:** 10.3389/fcimb.2016.00013

**Published:** 2016-02-09

**Authors:** Miguel A. Ares, José L. Fernández-Vázquez, Roberto Rosales-Reyes, Ma. Dolores Jarillo-Quijada, Kristine von Bargen, Javier Torres, Jorge A. González-y-Merchand, María D. Alcántar-Curiel, Miguel A. De la Cruz

**Affiliations:** ^1^Unidad de Investigación Médica en Enfermedades Infecciosas y Parasitarias, Hospital de Pediatría, Centro Médico Nacional Siglo XXI, Instituto Mexicano del Seguro Social, Hospital de PediatríaMexico City, Mexico; ^2^Departamento de Microbiología, Escuela Nacional de Ciencias Biológicas, Instituto Politécnico NacionalMexico City, Mexico; ^3^Unidad de Investigación en Medicina Experimental, Facultad de Medicina, Universidad Nacional Autónoma de MéxicoMexico City, Mexico; ^4^Cell Biology Institute, University of BonnBonn, Germany

**Keywords:** H-NS, *K. pneumoniae*, T3P, capsule, adherence, phagocytosis, virulence

## Abstract

*Klebsiella pneumoniae* is an opportunistic pathogen causing nosocomial infections. Main virulence determinants of *K. pneumoniae* are pili, capsular polysaccharide, lipopolysaccharide, and siderophores. The histone-like nucleoid-structuring protein (H-NS) is a pleiotropic regulator found in several gram-negative pathogens. It has functions both as an architectural component of the nucleoid and as a global regulator of gene expression. We generated a Δ*hns* mutant and evaluated the role of the H-NS nucleoid protein on the virulence features of *K. pneumoniae*. A Δ*hns* mutant down-regulated the *mrkA* pilin gene and biofilm formation was affected. In contrast, capsule expression was derepressed in the absence of H-NS conferring a hypermucoviscous phenotype. Moreover, H-NS deficiency affected the *K. pneumoniae* adherence to epithelial cells such as A549 and HeLa cells. In infection experiments using RAW264.7 and THP-1 differentiated macrophages, the Δ*hns* mutant was less phagocytized than the wild-type strain. This phenotype was likely due to the low adherence to these phagocytic cells. Taken together, our data indicate that H-NS nucleoid protein is a crucial regulator of both T3P and CPS of *K. pneumoniae*.

## Introduction

*Klebsiella pneumoniae* is an opportunistic Gram-negative bacterium belonging to the Enterobacteriaceae family causing nosocomial infections such as septicemia, pneumonia, urinary tract infections, surgical site infections and catheter-related infections (Han, 1995; Podschun and Ullmann, 1998; Schelenz et al., 2007; Ares et al., 2013). In addition, *K. pneumoniae* has been implicated in pyogenic liver abscesses in patients with meningitis, endophthalmitis and malignancies (Fung et al., 2002; Chang et al., 2008; Tsai et al., 2008; Alcántar-Curiel and Girón, 2015). Numerous nosocomial outbreaks caused by multiple-drug resistant *K. pneumoniae* have been reported (Nordmann et al., 2009; Hirsch and Tam, 2010; Schwaber et al., 2011). The main virulence determinants of *K. pneumoniae* are: capsular polysaccharide (CPS), lipopolysaccharide, siderophores, and pili (Gerlach et al., 1989; Podschun and Ullmann, 1998; Brisse et al., 2009). The *K. pneumoniae* genome codes for different pili such as Type 1 pili (T1P), Type 3 pili (T3P), and *E. coli* common pilus (ECP; Allen et al., 1991; Schurtz et al., 1994; Struve et al., 2009; Alcántar-Curiel et al., 2013). In contrast to T1P, the T3P can cause mannose-resistant agglutination of tannic acid-treated human erythrocytes (Podschun and Ullmann, 1998). The biogenesis of T3P is dependent on the *mrkABCDF* operon (Hornick et al., 1995; Huang et al., 2009). The filament is composed of the major pilus subunit MrkA and the tip adhesion protein MrkD (Gerlach et al., 1989). *K. pneumoniae* T3P mediate adherence to tracheal epithelial cells, renal tubular cells, basolateral surfaces of lung tissue and are crucial in biofilm formation (Tarkkanen et al., 1997; Sebghati et al., 1998; Langstraat et al., 2001; Jagnow and Clegg, 2003; Schroll et al., 2010). While the pili are required during the initial colonization of the host, the CPS impairs macrophage-mediated phagocytosis (Highsmith and Jarvis, 1985; Podschun and Ullmann, 1998; Alvarez et al., 2000). CPS is a complex layer of surface-associated polysaccharides which is important for the pathogenesis of *K. pneumoniae* in both, animal models as well as in infections of cultured cells (Cortés et al., 2002; Lawlor et al., 2005; Regueiro et al., 2006; March et al., 2013).

The histone-like nucleoid-structuring protein (H-NS) is a DNA-binding protein found in enteropathogens such as *Escherichia, Salmonella, Shigella, Vibrio*, and *Yersinia* (Tendeng and Bertin, 2003). It has functions as an architectural component of the nucleoid and as a global regulator of gene expression (Tendeng and Bertin, 2003; Dorman, 2004). It has been proposed that H-NS affects bacterial evolution by direct repression of AT-rich foreign DNA (i.e., pathogenicity islands) acquired by horizontal transfer events, to facilitate tolerance of these foreign sequences and to integrate them into a pre-existing regulatory network (Navarre et al., 2006, 2007; Dorman, 2007). Mutations in *hns*-like genes have pleiotropic effects in several bacteria and may cause defects in their growth or even bacterial cell death (Zhang et al., 1996; Tendeng et al., 2000; Heroven et al., 2004; Ellison and Miller, 2006; Lucchini et al., 2006; Navarre et al., 2006; Baños et al., 2008; Castang and Dove, 2012). Similar to other enterobacteria, *K. pneumoniae* is known to possess regions of horizontally acquired genetic sequences. However, there are no reports about the role of H-NS in this pathogen.

In this work we describe the effect of H-NS protein on the expression of both T3P and CPS, two of the main virulence determinants of *K. pneumoniae*. The absence of H-NS down-regulated the T3P and affected biofilm formation. In contrast, expression of CPS was derepressed in a Δ*hns* mutant, conferring a hypermucoviscous phenotype. Finally, the absence of H-NS resulted in low adherence to epithelial cells and macrophages and in high resistance to macrophage phagocytosis.

## Material and methods

### Bacterial strains and culture conditions

Bacterial strains and plasmids used in this study are listed in Table 1. Bacterial cultures were routinely grown in Luria-Bertani (LB) broth with or without antibiotics [200 μg/ml (ampicillin), 50 μg/ml (kanamycin), 30 μg/ml (chloramphenicol), or 10 μg/ml (tetracycline)] after overnight growth with shaking at 37°C.

**Table 1:** **Bacterial strains and plasmids used in this study**.

**Strain or plasmid**	**Genotype or description**	**References or source**
***K pneumoniae*** **STRAINS**		
Kpn 123/01	Wild-type, serotype K39	Clinical isolate
Kpn *hns*	Δ*hns*::Km^R^	This study
Kpn *mrkA*	Δ*mrkA*::Km^R^	This study
Kpn *cps*	Δ*cps*::Km^R^	This study
Kpn *hns cps*	Δ*hns*::Km^R^ Δ*cps*::Cm^R^	This study
***E. coli*** **K12 STRAIN**		
DH5α	Laboratory strain	Invitrogen
**PLASMIDS**		
pMPM-T3	p15A derivative low-copy-number cloning vector, *lac* promoter, Tc^R^	Mayer, 1995
pT3-H-NS	pMPM-T3 derivative expressing H-NS from the *lac* promoter	This study
pMPM-T6	p15A derivative cloning vector, pBAD (*ara*) promoter, Tc^R^	Mayer, 1995
pT6-MrkH	pMPM-T6 derivative expressing MrkH from the pBAD (*ara*) promoter	This study
pKD119	pINT-ts derivative containing the λ red recombinase system under an arabinose-inducible promoter, Tc^R^	Datsenko and Wanner, 2000
pKD4	pANTsγ derivative template plasmid containing the kanamycin cassette for λ Red recombination, Ap^R^	Datsenko and Wanner, 2000
pKD3	pANTsγ derivative template plasmid containing the chloramphenicol cassette for λ Red recombination, Ap^R^	Datsenko and Wanner, 2000

*Ap^R^, ampicillin resistance; Km^R^, kanamycin resistance; Cm^R^, chloramphenicol resistance; Tc^R^, tetracycline resistance*.

### Construction of *K. pneumoniae* mutants

*K. pneumoniae* 123/01 was isolated from a patient with pneumonia by bronchoalveolar washing. Capsular serotype K39 was determined by sequencing of *wzc* gene as previously described (Pan et al., 2013). *K. pneumoniae* was targeted for mutagenesis of *hns, mrkA* and *cps* following the procedure reported by Datsenko and Wanner (2000) with some modifications. Each purified PCR product was electroporated into competent *K. pneumoniae* carrying the lambda-Red recombinase helper plasmid pKD119, whose expression was induced by adding L-(+)-arabinose (Sigma) at a final concentration of 1.0%. For the Δ*cps* mutant, we deleted the chromosomal region from *galF* to *wzi* [Δ(*galF-orf2-wzi*)]. PCR fragments containing *hns, mrkA* and *cps* sequences flanking a kanamycin cassette were generated using gene-specific primer pairs (Table 2), and DNA of the pKD4 plasmid was used as template. For the Δ*hns* Δ*cps* double mutant, we generated a PCR fragment containing *cps* sequence flanking a chloramphenicol cassette using the pKD3 plasmid as template. The respective mutations were confirmed by PCR and sequencing.

**Table 2:** **Primers used in this study**.

**Primer**	**Sequence (5′-3′)**	**Target gene**	**RE**
**FOR qPCR**			
mrkA-5′	CCATGCAGCTGATACCAATG	*mrkA*	
mrkA-3′	GCAGCCTGGCAGTTAGAGAC		
fimA-5′	ACTGTTACCACCACCGAAGC	*fimA*	
fimA-3′	CTGGATACCGATGCCGATAC		
ecpA-5′	ACCTCGCGTCTTATCACCAA	*ecpA*	
ecpA-3′	CCGCTGATGATGGAGAAAG		
mrkH-5′	AAAATCAAACGCCTCACGAC	*mrkH*	
mrkH-3′	TGCGATGGGTCTGAATATGA		
mrkI-5′	CCAAGCGCAAAAAGAGAATC	*mrkI*	
mrkI-3′	AATAATCGTCTGGGCCAGTG		
mrkJ-5′	CGCTATTCGCGGTTATCACT	*mrkJ*	
mrkJ-3′	TATGATGGTTGCGCGATAAA		
fimB-5′	ATCGGATATCGACCTTGCTG	*fimB*	
fimB-3′	TAAAACTGTTGGCGGGAAAG		
fimE-5′	CAAAACGGACGCACTGTTTA	*fimE*	
fimE-3′	TGTCCCTCTTTCAGCCAGTT		
ecpR-5′	ATTTGGTCTGCCGATGACTC	*ecpR*	
ecpR-3′	ATTTGGTCTGCCGATGACTC		
rcsA-5′	ACGGTATCGTCGCATAAAGG	*rcsA*	
rcsA-3′	AGGTGATGTTTTCGGTCAGC		
galF-5′	CAAAGGCAATTCCAAAGGAG	*galF*	
galF-3′	TGCGTCACCAGAACAATCTC		
wzi-5′	CAGGGGTTTGGTCAGACACA	*wzi*	
wzi-3′	CGTTGAAGCGTGATCCGTTG		
manC-5′	AGCGGCATGTTTATGTTCCG	*manC*	
manC-3′	AAATGTCATGCGGGATGCTG		
rrsH-5′	CAGCCACACTGGAACTGAGA	*rrsH*	
rrsH-3′	GTTAGCCGGTGCTTCTTCTG		
**FOR GENE CLONING**			
hns-XhoI-5′	GGG*CTCGAG*TATTAGTTCAACAAA CCACCCCAC	*hns*	*Xho*I
hns-EcoRI-3′	GGG*GAATTC*GGCAAAAAAAATCCC GCCGCAGCGGG		*Eco*RI
mrkH-NcoI-5′	AAG*CCATGG*ATATGACAGAGGGAA CGATAAAG	*mrkH*	*Nco*I
mrkH-HindIII-3′	CCA*AAGCTT*TCAATGATGATGATGATG ATGGATTCTCTTTTTGCGCTTGGCTTC		*Hind*III
**FOR GENE DELETIONS**			
Kpn-hns-H1PI	TATAAGTTTGAGATTACTACAATGAG CGAAGCACTTAAAATTTTGTGTAGGC TGGAGCTGCTTCG	*hns*	
Kpn-hns-H2P2	TTTTATAGCGATCAACGGAGATTAGA TCAGGAAATCGTCCAGTGACATATGA ATATCCTCCTTAG		
Kpn-mrkA-H1P1	CACTCTGACAAGGAAATGGCAATGAA AAAGGTTCTTCTCTCTGCATGTAGGCT GGAGCTGCTTCG	*mrkA*	
Kpn-mrkA-H2P2	CAGTTTTATTTTCTGACGGAATTACTG GTAAGTAATTTCGTAAGTCATATGAAT ATCCTCCTTAG		
Kpn-cps-H1P1	TTTGCAACCAAACAGGTGAAGATGAA TATGGCGAATTTGAAAGCGTGTAGGCT GGAGCTGCTTCG	*cps*	
Kpn-cps-H2P2	CCCTGTTTTCAGCATTCAGCCTTATAA ACTAAACGGTATTTCTATCATATGAAT ATCCTCCTTAG		

*Italic letters indicate the respective restriction enzyme site in the primer. The sequence corresponding to the template plasmids pKD4 or pKD3 is underlined. RE, restriction enzyme for which a site was generated in the primer*.

### Construction of plasmids

The pT3-H-NS plasmid was generated by cloning a PCR product containing the corresponding *hns* region of *K. pneumoniae* into the pMPM-T3 plasmid (see primers in Table 2). The PCR product was digested with *Xho*I and *Eco*RI and ligated into pMPM-T3 digested with the same enzymes. pT6-MrkH was constructed by cloning a PCR product containing the *mrkH* region, which was digested with *Nco*I and *Hind*III and ligated into pMPM-T6 (see Table 2). The identities of the inserts were confirmed by DNA sequencing.

### Quantitative RT-PCR

Total RNA extraction was performed using the hot phenol method as previously described (Jahn et al., 2008). DNA was removed with TURBO DNA-free (Ambion, Inc.) and the quality of RNA was assessed using a NanoDrop (ND-1000; Thermo Scientific) and an Agilent 2100 bioanalyzer with a Picochip (Agilent Technologies). The absence of contaminating DNA was controlled by lack of amplification products after 35 qPCR cycles. cDNA was prepared using 1 μg of RNA, random hexamer primers (0.2 μg/μl), and M-MulV-RT (20 U/μl, reverse transcriptase of Moloney Murine leukemia Virus; Thermo Scientific). Specific primers were designed with the Primer3Plus software (http://www.bioinformatics.nl/cgi-bin/primer3plus/primer3plus.cgi/) and are listed in Table 2. For LightCycler reactions, a master mix of the following components was prepared: 3.0 μl PCR-grade water, 1.0 μl (10 μM) forward primer, 1.0 μl (10 μM) reverse primer, 10 μl 2x SYBR Green I Master Mix, and 5.0 μl cDNA (50–100 ng). A multiwell plate was sealed with sealing foil, centrifuged at 1500 *g* for 2 min and loaded into the LightCycler 480 instrument (Roche). Amplification was performed in triplicate wells for each sample analyzed. Control reactions with no template (water) and minus-reverse transcriptase (RNA) were run with all reactions. Real-time PCR analysis was performed using the following conditions: denaturation (95°C for 10 min); amplification and quantification repeated for 45 cycles (95°C for 10 s, 57°C for 20 s, 72°C for 30 s with a single fluorescence measurement); melting curve (95°C for 10 s, 65°C for 1 min with continuous fluorescence measurement at 97°C); and finally a cooling step at 40°C for 10 s. Melting curve analysis was performed after each run to confirm specificity of the primers. 16S rRNA was used as a reference gene for normalization and the relative gene expression was calculated using the 2^−ΔCt^ method (Livak and Schmittgen, 2001).

### Mucoviscosity

The mucoviscosities of *K. pneumoniae* strains were determined by a string test and measured by centrifugation as described previously (Pan et al., 2011; Lin et al., 2012). The string test was performed stretching a colony that had been grown overnight on a blood agar plate, using a loop. To further measure the levels of mucoviscosity, a low speed centrifugation was performed. Briefly, equal numbers of exponential phase-cultured bacteria were centrifuged at 1000 *g* for 5 min. The supernatant was subjected to measurement of the absorbance at 600 nm.

### Glucuronic acid analysis

Capsular polysaccharides were extracted and quantified using a colorimetric assay for glucuronic acid as previously described (Lin et al., 2009). Basically, 500 μl of bacterial cultures were mixed with 100 μl of 1% zwittergent 3–14 in 100 mM citric acid and then the mixtures were incubated at 50°C for 20 min. After centrifugation, 250 μl of supernatants were transferred into new tubes, and 1 ml of absolute ethanol was added to precipitate the CPS. The pellets were dissolved in 200 μl of distilled water, and then 1200 μl of 12.5 mM borax in concentrated H_2_SO_4_ were added. The mixtures were vigorously vortexed, boiled for 5 min, and then cooled. 20 μl of 0.15% 3-hydroxydiphenol in 0.5% NaOH were added to the mixture and the absorbance was measured at 520 nm. The glucuronic acid concentration in each sample was determined from a standard curve of glucuronic acid and expressed in micrograms/10^9^ CFU.

### Adherence assays to cultured eukaryotic cells

Monolayers of HeLa (ATCC CCL-2) human cervix epithelial and A549 (ATCC CCL-185) human lung epithelial cell lines (7 × 10^5^) were infected with the indicated strains of an LB broth overnight culture at a multiplicity of infection (MOI) of 100. Epithelial cells were grown in DMEM (Invitrogen) with 10% fetal bovine serum (FBS). After infection, eukaryotic cells were incubated in DMEM with no FBS for 2 h at 37°C under an atmosphere of 5% CO_2_. After the 2 h incubation period, cells were rinsed three times with PBS to remove unbound bacteria. For quantification of adherence, the cells were lysed with a solution of 0.1% Triton X-100. After homogenization, 10-fold serial dilutions were plated onto LB agar plates to determine total CFUs. The results shown are the mean of at least three experiments performed in triplicate on different days.

### Phagocytosis of bacteria by macrophages

THP-1 (ATCC TIB-202) human monocytes (differentiated to macrophages with 200 nM of phorbol 12-myristate 13-acetate for 24 h) and RAW264.7 (ATCC TIB-71) murine macrophages (6 × 10^5^) were seeded into 24-well tissue culture plates. Bacteria were grown in 5 ml of LB broth to the exponential phase. Macrophages were infected with a MOI of 100 in a final volume of 1 ml RPMI 1640 tissue culture medium supplemented with 10% heat-inactivated FBS. To synchronize infection, plates were centrifuged at 200 *g* for 5 min. Plates were incubated at 37°C under an humidified 5% CO_2_ atmosphere. After 2 h, cells were rinsed three times with PBS and incubated for an additional 60 min with 1 ml of RPMI 1640 containing 10% FBS and gentamicin (100 μg/ml) to eliminate extracellular bacteria. Cells were then rinsed again three times with PBS and lysed with 0.1% Triton X-100. After homogenization, 10-fold serial dilutions were plated onto LB agar plates to determine total CFUs. Adherence of *K. pneumoniae* strains (grown to the exponential phase) to macrophages was performed as previously described (Rosales-Reyes et al., 2012), incubating 1 h at 4°C to inhibit phagocytosis.

### Biofilm formation assay on abiotic surface

Adhesion to abiotic surface (polystyrene) was analyzed using 96-well plates as described previously (Saldaña et al., 2014). Overnight cultures of bacteria grown in LB broth (10 μl) were added to 1 ml of LB. This volume was distributed in quintuples (100 μl per well) into a 96-well plate and incubated at room temperature for 24 h. Unbound bacteria were removed by washing the wells three times with PBS, and bound bacteria were stained with 1% crystal violet (CV) for 20 min. Wells were thoroughly rinsed three times with PBS, and the dye was solubilized in 100 μl of ethanol 70%. Finally, the amount of extracted crystal violet was determined by measuring the OD_600_ using an enzyme-linked immunosorbent assay (ELISA) Multiskan plate reader.

### Statistical analysis

For statistical differences, one-way ANOVA followed by the Tukey's comparison test was performed using Prism 5.0 (GraphPad Software Inc., San Diego, CA, USA). *P* ≤ 0.05 was considered statistically significant.

## Results

### Generation of an *hns* mutant of *K. pneumoniae*

**Figure 1:**
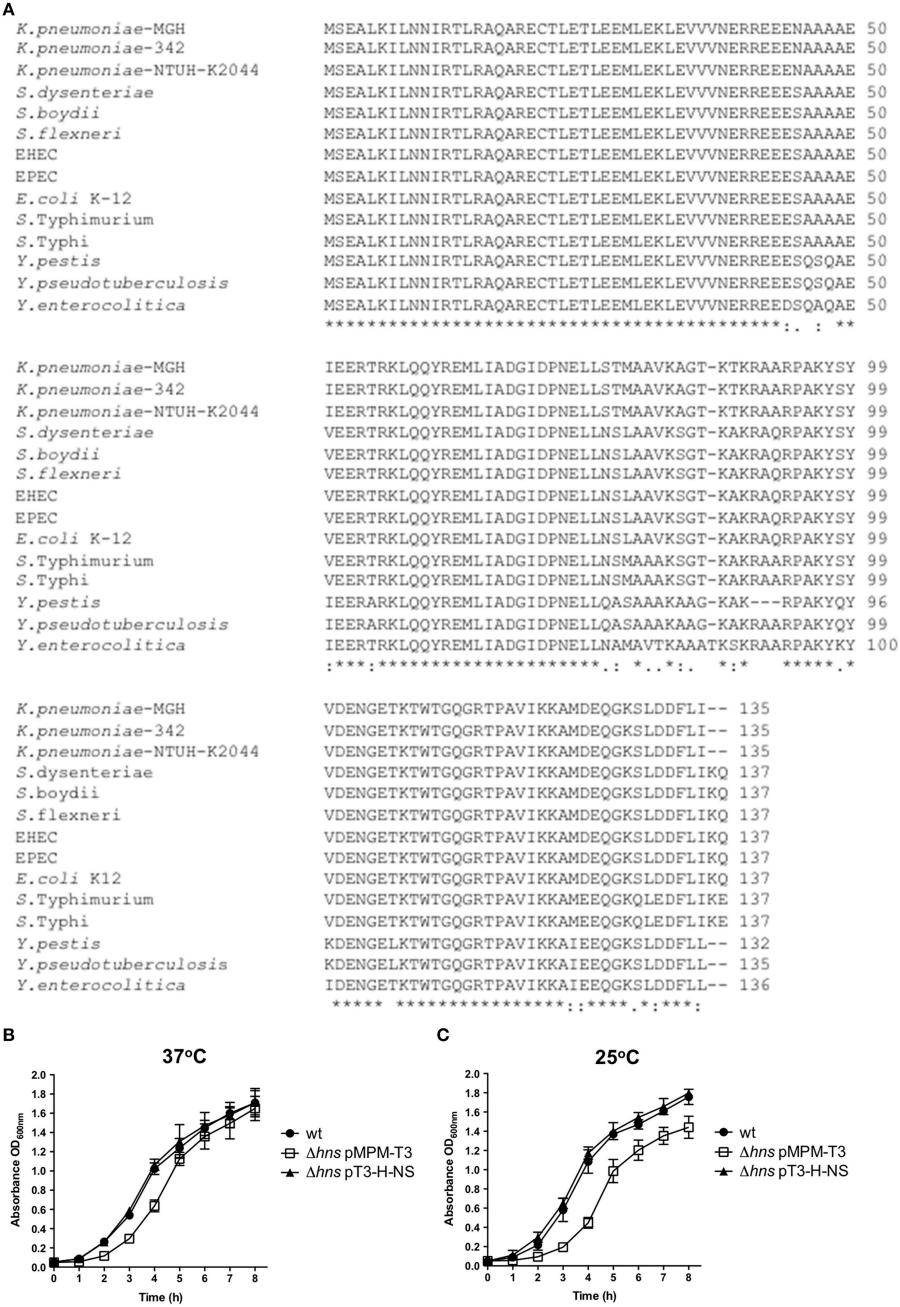
**H-NS protein in ***K. pneumoniae***. (A)** Alignment of amino acid sequences of H-NS proteins from several enterobacteria: *K. pneumoniae* (MGH, 342 and NTUH-K2044), *Shigella dysenteriae* (Sd197), *Shigella boydii* (Sb227), *Shigella flexneri* (2a str. 2457T), Enterohemorragic *E. coli* (EDL933), Enteropathogenic *E. coli* (E2348/69), *E. coli* K-12 (MG1655), *Salmonella enterica* serovar Typhimurium (LT2), *Salmonella enterica* serovar Typhi (CT18), *Yersinia pestis* (KIM 10), *Yersinia pseudotuberculosis* (IP 32953) and *Yersinia enterocolitica* (8081). Analysis was performed using the ClustalW2 software (http://www.ebi.ac.uk/Tools/msa/clustalw2/). Growth kinetics of wild-type *K. pneumoniae*, and the isogenic mutants at 37°C **(B)** and 25°C **(C)**. Bacterial cultures were grown for 8 h in LB medium.

H-NS amino acid sequences of *K. pneumoniae* strains were homologous to H-NS proteins of enteric bacteria such as *Salmonella, Yersinia, Shigella*, and *E. coli* (Figure 1A). The *hns* gene of the strain 123/01 used in this study was completely identical to all *K. pneumoniae* sequenced strains (data not shown). By using the λ-red homolog recombinase (Datsenko and Wanner, 2000), we were able to replace the *hns* gene of the *K. pneumoniae* genome with a kanamycin resistance cassette. The *hns* gene of *K. pneumoniae* was cloned into a plasmid yielding pT3-H-NS to complement the Δ*hns* mutant. In terms of resistance to antibiotics, the Δ*hns* mutant did not differ with respect to the wild type strain (data not shown). The growth in LB broth of *K. pneumoniae* strains was followed over a period of 8 h at 37 and 25°C. Growth of the Δ*hns* mutant was slightly attenuated at 37°C, mainly in the exponential phase but reaching the stationary phase like the wild-type strain (Figure 1B). In contrast, at 25°C the Δ*hns* mutant did not reach the growth of the wild-type strain in the stationary phase (Figure 1C). Growth of the complemented strain harboring pT3-H-NS was restored to wild-type levels at both temperatures. This observation indicates that H-NS is required for optimal growth of *K. pneumoniae* as has been shown for other enterobacteria (Zhang et al., 1996; Tendeng et al., 2000; Heroven et al., 2004; Ellison and Miller, 2006; Lucchini et al., 2006; Navarre et al., 2006; Baños et al., 2008; Castang and Dove, 2012).

### H-NS differentially regulates the fimbrial repertoire of *K. pneumoniae*

**Figure 2:**
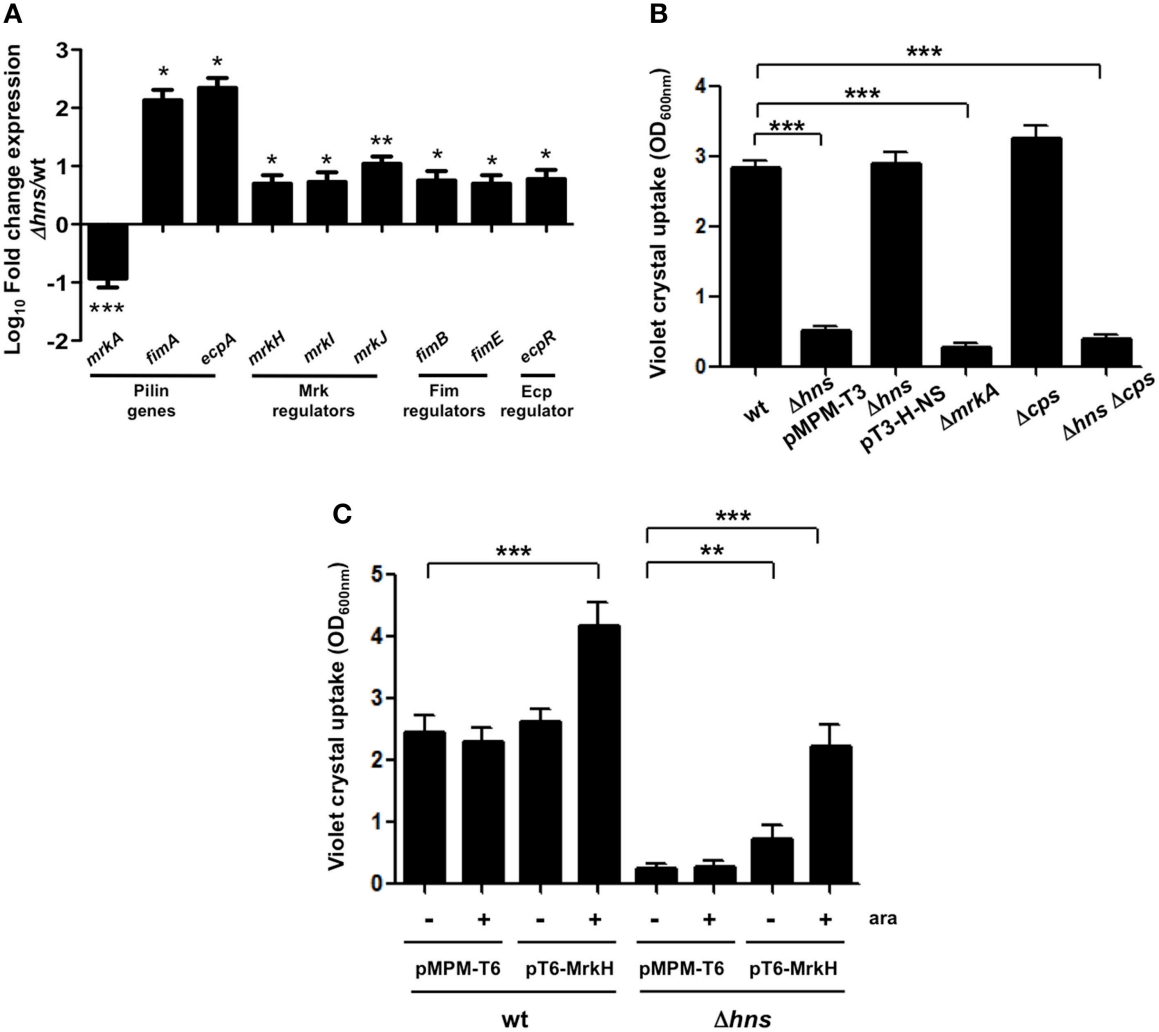
**H-NS positively regulates ***mrkA*** expression. (A)** Fold-change expression (qRT-PCR) of the pilin genes and their regulators in the Δ*hns* mutant as compared to the *K*. *pneumoniae* wild-type strain. **(B)** Quantification of biofilm formation by measuring Crystal Violet uptake. **(C)** Quantification of biofilm formation by measuring Crystal Violet uptake overexpressing the MrkH activator protein (0.1% arabinose) in both wild-type and *hns* background. Results shown represent mean and standard deviations of 3 independent experiments performed. ns, not significant; statistically significant with respect to the wild-type strain ^***^p < 0.001; ^**^p < 0.01; ^*^p < 0.05.

We reported previously that *ecpA, mrkA* and *fimA* fimbrial genes are highly prevalent in *K. pneumoniae* strains (Alcántar-Curiel et al., 2013). To demonstrate the role of H-NS in regulation of fimbrial genes, we determined transcriptional expression levels of *ecpA, mrkA* and *fimA* by qRT-PCR. Both, *ecpA* and *fimA* were derepressed in the absence of H-NS. In contrast to *ecpA* and *fimA, mrkA* was repressed in the absence of H-NS (Figure 2A). In addition to pilin genes themselves, we evaluated the expression of genes regulating expression of three pili types. Regulatory genes for the pilins analyzed were up-regulated in the absence of H-NS. The *mrkJ* gene codes for a phosphodiesterase (Johnson and Clegg, 2010) and negatively regulates *mrkA* expression by degrading c-di-GMP, thereby inhibiting MrkH activity, which is the central activator of T3P (Wilksch et al., 2011). To corroborate decreased expression of the *mrkA* pilin gene in absence of H-NS, we performed biofilm formation assays. Biofilm formation in *K. pneumoniae* is MrkA-dependent (Langstraat et al., 2001; Wilksch et al., 2011). Indeed, the absence of MrkA reduced *K. pneumoniae* biofilm formation by 10-fold (Figure 2B). The Δ*hns* mutant was impaired in biofilm formation (5-fold) similar to the Δ*mrkA* mutant, while this phenotype was counteracted by complementing the Δ*hns* mutant with the pT3-H-NS plasmid (Figure 2B). In addition to T3P, the capsule polysaccharide (CPS) is a crucial virulence determinant in *K. pneumoniae*. To evaluate the role of CPS in biofilm formation, we assayed the Δ*cps* and Δ*hns* Δ*cps* mutants. The Δ*cps* mutant was not affected in biofilm formation. However, the Δ*hns* Δ*cps* double mutant was impaired in biofilm formation similar to the Δ*hns* single mutant (Figure 2B). These observations suggest that CPS has no role in biofilm formation and that downregulation of T3P expression is the main reason for the biofilm phenotypes observed in the Δ*hns* mutant. To corroborate that the effect of H-NS on biofilm formation is due to the transcriptional repression of *mrkA* and not due to overexpression of the capsule possibly resulting in steric hindrance of T3P, we generated wild type and Δ*hns* mutant strains overexpressing the MrkH activator protein. Indeed, MrkH overexpression positively affected biofilm formation (~2-fold) in the wild-type strain (Figure 2C). Interestingly, overexpression of the MrkH protein counteracted the decrease in biofilm formation observed in the Δ*hns* mutant (~9-fold) regardless of the excess production of CPS (Figure 2C). These data corroborated H-NS as a positive regulator of TP3 gene expression.

### The absence of H-NS results in a hypermucoviscous phenotype of *K. pneumoniae*

**Figure 3:**
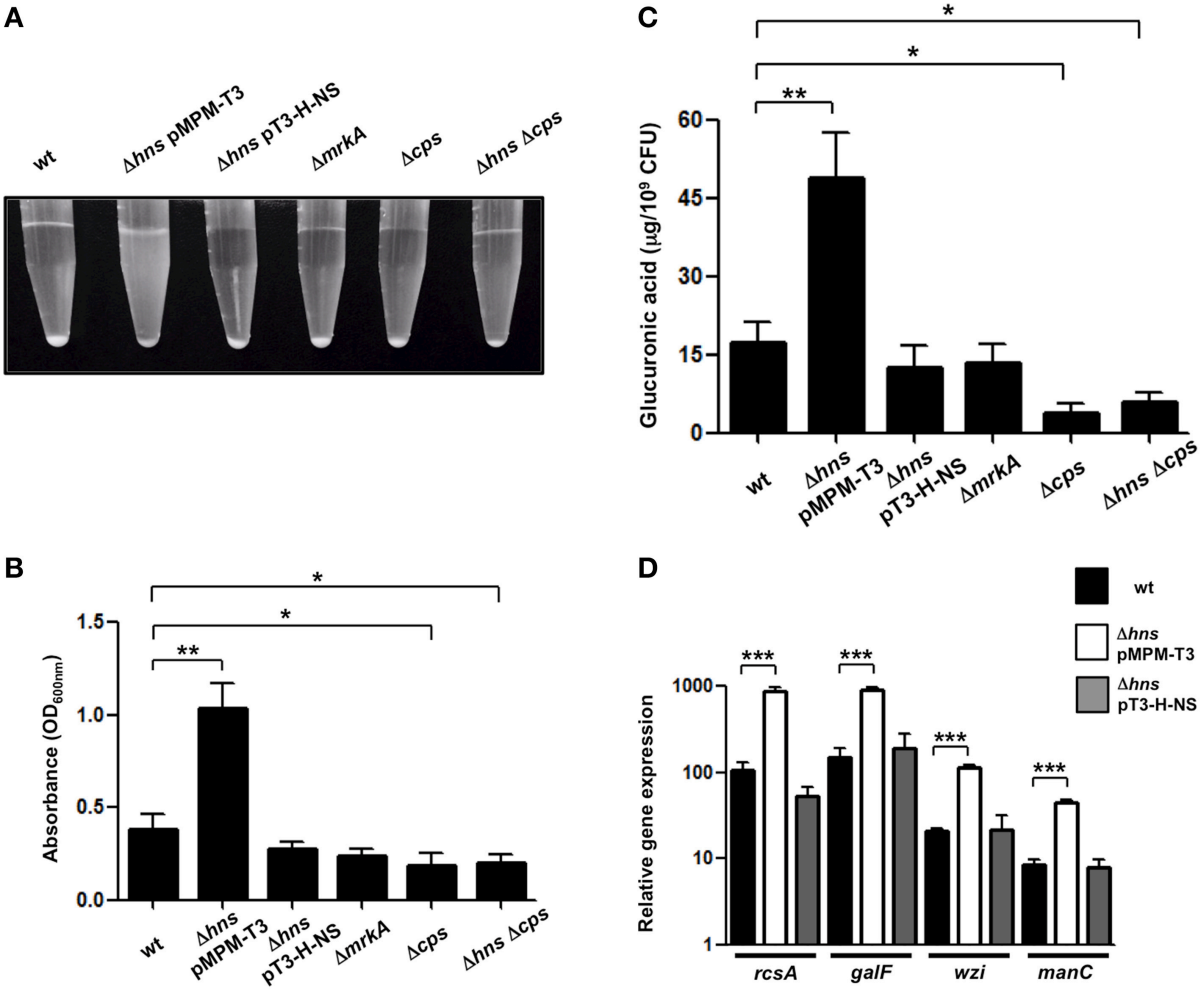
**H-NS represses capsular polysaccharide in ***K. pneumoniae***. (A,B)** Mucoviscosity of *K. pneumoniae* wild-type, Δ*hns* mutant, complemented Δ*hns* mutant, Δ*mrkA* mutant, Δ*cps* mutant, and Δ*hns* Δ*cps* mutant. The mucoviscosity was determined by low speed centrifugation and is expressed as OD_600_ of the supernatant. **(C)** Capsule quantification of *K. pneumoniae* strains. The glucuronic acid concentration in each strain was determined from capsular polysaccharides extracted of 0.5 ml bacterial cultures. **(D)** Transcriptional expression (qRT-PCR) of the *rcsA, galF, wzi, and manC* genes in the WT *K*. *pneumoniae* strain, Δ*hns* mutant and complemented Δ*hns* mutant. Data represent the mean of at least three independent experiments (mean ± SD). Statistically significant with respect to the wild-type strain ^***^p < 0.001; ^**^p < 0.01; ^*^p < 0.05.

The Δ*hns* mutant colony morphology was hypermucoviscous compared to wild-type bacteria on agar plates while the complemented strain was similar to the wild-type strain (data not shown). To determine the levels of mucoviscosity, we measured the supernatant of suspensions of wild-type, Δ*hns* mutant and complemented Δ*hns* mutant bacteria centrifuged at low speed. Indeed, the absence of H-NS resulted in higher mucoviscosity levels compared to the wild-type strain (~3-fold), while complementation restored the wild-type phenotype (Figures 3A,B). To determine the amount of CPS quantitatively, we performed a biochemical assay, which measured the capsular glucuronic acid. As shown in Figure 3C, the CPS level increased in the absence of H-NS (~3-fold), while the complemented Δ*hns* strain had similar amounts of CPS compared to the wild-type strain. The absence of MrkA did not alter the CPS production; however in both the Δ*cps* and Δ*hns* Δ*cps* mutants the CPS amount was considerably diminished (~4-fold), indicating that increasing of the capsule in the *hns* background is dependent on capsular genes. Genetically, capsule-generating genes of *K. pneumoniae* are encoded in the *cps* cluster, including three transcriptional units, being *galF, wzi*, and *manC* the first genes for each promoter (Chou et al., 2004; Chuang et al., 2006; Pan et al., 2011). *K. pneumoniae* strains belonging to serotype K39 contain the three capsule-generating genes described above (Pan et al., 2013, 2015). In addition, *cps* genes are activated by the RcsA regulatory protein (Wehland and Bernhard, 2000; Lin et al., 2011, 2013). Using qRT-PCR we monitored the expression of *rcsA, galF, wzi*, and *manC* in wild-type *K. pneumoniae*, the Δ*hns* mutant and the complemented Δ*hns* mutant strain. Expression levels of *rcsA, galF, wzi*, and *manC* were derepressed ~6 to 8-fold in the absence of H-NS (Figure 3D). The complemented Δ*hns* mutant presented expression levels similar to the wild-type strain. These observations supported an inhibitory effect of H-NS on the expression/production of a polysaccharide capsule in *K. pneumoniae*.

### Fimbrial and capsular genes of *K. pneumoniae* are thermoregulated by H-NS

**Figure 4:**
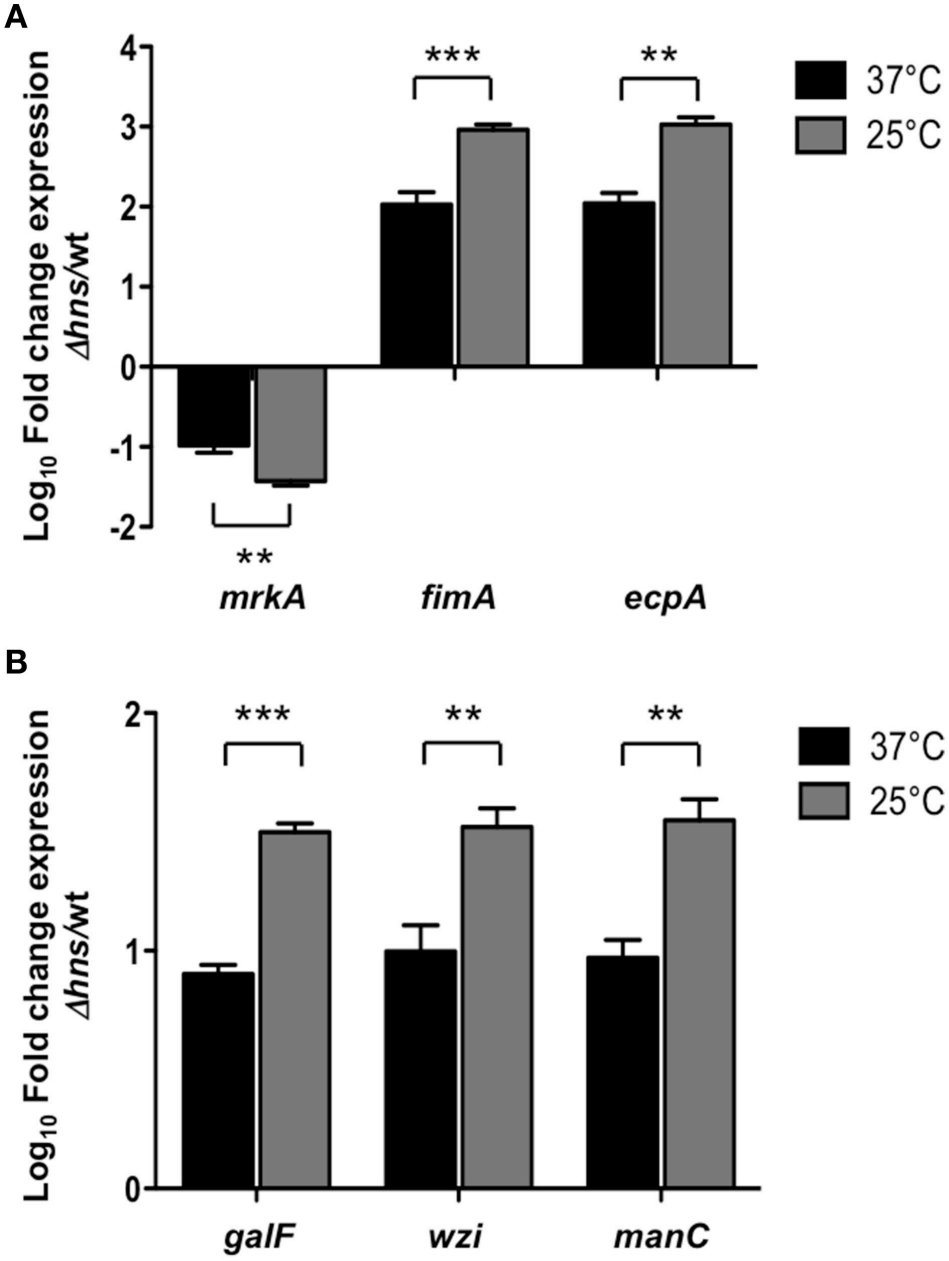
**H-NS thermoregulates both fimbrial and capsular genes**. Fold-change expression (qRT-PCR) of the fimbrial **(A)** and capsular genes **(B)** in the Δ*hns* mutant as compared to the *K*. *pneumoniae* wild-type strain. Data represent the mean of at least three independent experiments (mean ± SD). Statistically significant with respect to the wild-type strain ^***^p < 0.001; ^**^p < 0.01.

H-NS nucleoid protein has been reported to be an essential component in thermoregulation of virulence factors in several pathogenic bacteria (Falconi et al., 1998; Umanski et al., 2002; Ono et al., 2005; Duong et al., 2007). To analyze if the repressor effect of H-NS was temperature-dependent, we performed qRT-PCR experiments determining the transcriptional expression of both fimbrial (*mrkA, fimA, ecpA*) and capsule-generating (*galF, wzi, manC*) genes in the wild-type and Δ*hns* mutant at 37 and 25°C. At 25°C, the repressor effect of H-NS was higher than at 37°C, specifically 3.33-, 8.72-, and 9.66-fold for *mrkA, fimA*, and *ecpA*, respectively (Figure 4A). For capsular genes, H-NS-mediated repression was about 4-fold higher at 25°C compared to 37°C (Figure 4B). These data clearly indicate that at low temperatures (25°C), H-NS efficiently represses both fimbrial and capsular genes as compared to 37°C by maintaining down-regulation of these virulence genes.

### H-NS is involved in *K. pneumoniae* adherence to and phagocytosis by eukaryotic cells

**Figure 5:**
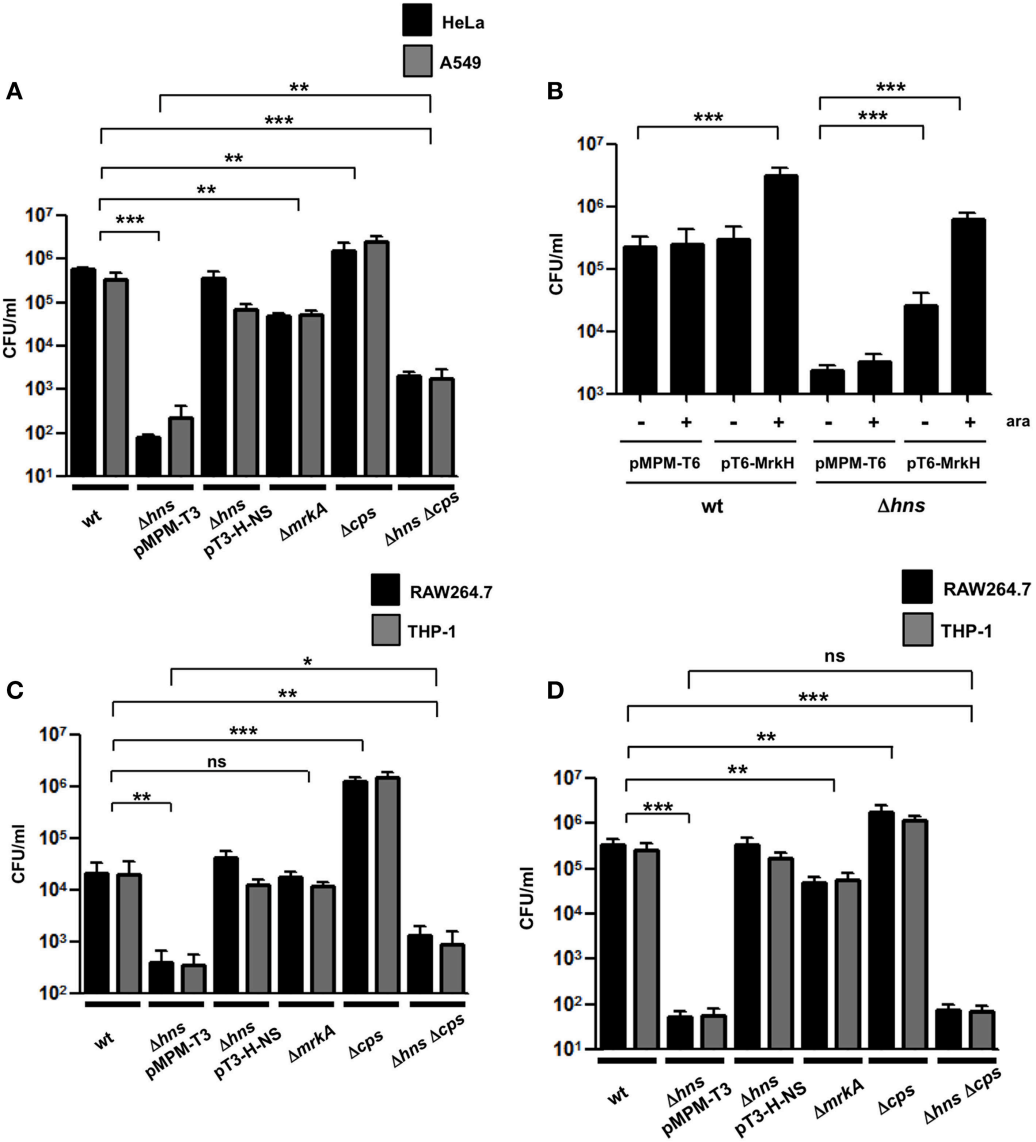
**Adherence and phagocytosis of the ***K. pneumoniae*** Δ***hns*** mutant. (A)** Comparison of adherence levels of *K. pneumoniae* wild-type, Δ*hns* mutant, complemented Δ*hns* mutant, Δ*mrkA* mutant, Δ*cps* mutant, and Δ*hns* Δ*cps* mutant to HeLa and A549 cells. **(B)** Adherence levels of wild-type strain and isogenic Δ*hns* mutant to HeLa cells, overexpressing the MrkH activator protein (0.1% arabinose). **(C)** Comparison of phagocytic uptake of indicated *K. pneumoniae* strains by RAW264.7 and THP-1 macrophages. **(D)** Adherence levels of *K. pneumoniae* wild-type strain and isogenic mutants to RAW264.7 and THP-1 macrophages. Results represent means and standard deviations of the results obtained from the 3 experiments performed in triplicates. ns, not significant; statistically significant with respect to the wild-type strain ^***^p < 0.001; ^**^1*p* < 0.01; ^*^p < 0.05.

*K. pneumoniae* is able to adhere to different epithelial cell lines such as A549 and HeLa (Moranta et al., 2010; Alcántar-Curiel et al., 2013). We evaluated the adherence of the *K. pneumoniae* wild-type bacteria, the Δ*hns* mutant, the complemented Δ*hns* mutant, the Δ*mrkA* mutant, the Δ*cps* mutant and the Δ*hns* Δ*cps* double mutant to A549 or HeLa cells. Surprisingly, the absence of H-NS dramatically impaired the adherence of *K. pneumoniae* to both, A549 and HeLa cells (~4500-fold), while the complemented Δ*hns* mutant showed an adherence that was similar to that of the wild-type strain (Figure 5A). Interestingly, the Δ*mrkA* mutant was less adherent than the wild-type strain (~9-fold) but not comparable to the Δ*hns* mutant, suggesting that the phenotype observed with the Δ*hns* mutant cannot be explained solely by the decrease of MrkA expression (Figure 5A). Similar to the biofilm assay, we overexpressed the MrkH activator protein to exclude possible effects of capsule-mediated steric hindrance on T3P activity. Overexpression of MrkH enhanced the adherence of *K. pneumoniae* to HeLa cells for both, wild-type bacteria (~14-fold) and Δ*hns* mutant (~263-fold) (Figure 5B), supporting the notion that H-NS affects the adherence by transcriptional control of *mrkA* pilin. The absence of CPS resulted in a slight increase in adherence of *K. pneumoniae* to both epithelial cell types (~5-fold). Bacteria deficient in both *hns* and *cps* genes adhered in higher numbers to epithelial cells compared to those deficient in *hns* alone (~15-fold). However, the levels of adherence of the Δ*hns* Δ*cps* double mutant did not reach the numbers of the wild-type strain, indicating that CPS is partially involved in the low adherence observed with the Δ*hns* single mutant (Figure 5A). To discard that biofilm formation on plastic surfaces did not affect the adherence assays during 2 h of incubation, we quantified the CFU/ml of adhered bacteria on plastic wells at this time with no eukaryotic cells. We were unable to find adhered bacteria on the plastic surface during 2h. Furthermore, biofilm formation assays were performed and all strains examined did not form biofilm at 2 h of incubation in the conditions examined for eukaryotic cells (DMEM or RPMI, 37°C, 5% CO_2_). A crucial event in the pathogenesis of *K. pneumoniae* is the evasion of macrophage phagocytosis. We used THP-1 differentiated macrophages and RAW264.7 cells to evaluate the effect of H-NS deletion on phagocytosis. A Δ*hns* mutant was considerably less phagocytized by macrophages (~55-fold) and the complemented strain was recovered in numbers similar to the wild-type strain (Figure 5C). Bacterial growth rates during adherence and phagocytosis were similar in the different strains analyzed (data not shown), indicating that the low levels of phagocytosis observed with the Δ*hns* mutant were not due to growth defects. The absence of MrkA did not alter the phagocytosis of *K. pneumoniae* by macrophages, while the absence of CPS increased the phagocytosis by both cell lines (~67-fold). In contrast, a Δ*hns* Δ*cps* double mutant was phagocytosed in similar bacterial numbers as the Δ*hns* single mutant. To determine if the low level of phagocytosis showed by the Δ*hns* Δ*cps* double mutant was due to the initial stage of adherence, we performed adherence assays using both macrophages cell lines. The Δ*hns* and the Δ*hns* Δ*cps* mutants presented the same low levels of adherence to macrophages, indicating that the evasion of phagocytosis is mainly due to impaired adherence (Figure 5D). These observations suggest that the H-NS nucleoid protein in *K. pneumoniae* is relevant for both, adherence to and phagocytosis by eukaryotic cells.

## Discussion

H-NS is a pleiotropic regulator, which modulates expression of virulence determinants of several enteropathogens (Dorman, 2004). This study describes for the first time the role of H-NS in expression of *K. pneumoniae* virulence features. The *K. pneumoniae* H-NS protein is homologous to other H-NS-like proteins of different enteropathogens such as *E. coli, Salmonella, Yersinia*, and *Shigella*. In terms of bacterial fitness, the absence of H-NS resulted in a slight defect in *K. pneumoniae* growth in LB broth at 37°C, but more evident at low temperature such as 25°C. As previously reported in other *hns* mutants, this could be due to dysregulated expression of non-related genes (Navarre et al., 2007). Pili are relevant for the adherence to host cells as well as in biofilm formation (Podschun and Ullmann, 1998; Langstraat et al., 2001; Wilksch et al., 2011). Interestingly, a Δ*hns* mutant differentially regulated fimbrial genes, as we observed an increase of both *fimA* and *ecpA* and a repression of *mrkA* expression. FimA and EcpA pilin subunits were previously reported to be repressed by H-NS in *E. coli*, showing similar regulation for pilin genes as for regulatory genes in both bacteria (Dorman and Ní Bhriain, 1992; Schembri et al., 1998; Martínez-Santos et al., 2012; Lehti et al., 2013). The *mrkA* expression was down-regulated in the absence of H-NS, likely by up-regulation of *mrkJ*, which is a negative regulator of T3P (Johnson and Clegg, 2010), albeit MrkH and MrkI transcriptional regulators were also derepressed. In the absence of H-NS, high levels of MrkJ may degrade c-di-GMP affecting the MrkH activity as transcriptional activator of the *mrkA* gene (Wilksch et al., 2011). The positive effect of H-NS on *mrkA* suggests a post-transcriptional process indirectly affecting MrkA expression, as has been previously described for other genes regulated by H-NS (Bertin et al., 1994; Suzuki et al., 1996; Johansson et al., 1998; Park et al., 2010). The positive role of H-NS on *mrkA* expression was corroborated by the fact that the Δ*hns* mutant was impaired in biofilm formation similarly to the mutant deficient in *mrkA*. Since pili are important in the early stage of infection, we analyzed the contribution of H-NS and Mrk to the adherence to human epithelial cells. A Δ*hns* mutant was dramatically affected in the adherence to both, A549 and HeLa cells, while the absence of MrkA led to a significant but comparatively mild decrease in adherence. T3P have been expressed in an *E. coli* background (Tarkkanen et al., 1997), yet this is the first report about the contribution of MrkA pilin to the adherence to A549 and HeLa epithelial cells using a Δ*mrkA* mutant. The low adherence levels of the Δ*hns* mutant could not be observed with the Δ*mrkA* mutant, suggesting that this decrease in adherence to human epithelial cells may be caused by different factors. In addition to transcriptional repression, hypermucoviscosity observed in the Δ*hns* mutant could block the exposition of Mrk pili and therefore interfere with their attachment to the abiotic surface. Previous studies showed that T1P function can be inhibited by the presence of the capsule by steric overcrowding and it has been suggested that this would also affect exposition of Mrk pili (Schembri et al., 2005; Wilksch et al., 2011). Overexpression of MrkH activator protein showed, however, that T3P are not affected by capsule-mediated steric hindrance in the *hns* background neither with respect to biofilm formation, nor during adherence to epithelial cells. Hypervirulent *K. pneumoniae* strains produce large amounts of CPS, which confer both, a mucoviscous phenotype and resistance to phagocytosis (Lin et al., 2004; Regueiro et al., 2006). We found that the Δ*hns* mutant was hypermucoviscous with respect to the wild-type strain. This increase in CPS correlated with the derepression of capsular genes (Chuang et al., 2006; Ho et al., 2011; Lin et al., 2013). Controversial results regarding the involvement of capsule on biofilm formation in *K. pneumoniae* have been reported (Schembri et al., 2005; Boddicker et al., 2006; Balestrino et al., 2008; Wu et al., 2011; Wang et al., 2015). However, our data support observations stating that CPS is not related with biofilm formation.

As to CPS regulation by H-NS, a mucoid morphology in an *E. coli hns* background has been shown in previous reports (Ebel and Trempy, 1999). This phenotype was due to up-regulation of *rcsA*, which activates the *cps* locus (Sledjeski and Gottesman, 1995). Interestingly, we observed the same phenomenon in *K. pneumoniae*, since capsular structural genes (*galF, wzi*, and *manC*) and a capsular regulator (*rcsA*) were derepressed in the absence of H-NS. Moreover, the *K. pneumoniae* Δ*hns* mutant was phagocytized in lower numbers as the wild-type strain, likely related to its high mucoviscosity which confers resistance to phagocytic uptake by macrophages (Williams et al., 1983; Cortés et al., 2002). Surprisingly, uptake of bacteria deficient in both, *hns* and *cps* was similar to that of mutants deficient in *hns* alone. This decrease was likely due to impaired adherence to macrophages, indicating that H-NS regulates the initial stages of *K. pneumoniae* recognition by phagocytic cells. Our data show that there are probably others factors involved in macrophage adherence in addition to CPS. Pili of gram-negative bacteria such as *E. coli* T1P, *Porphyromonas gingivalis* FimA (also called T2P), Gram-positive *Lactobacillus rhamnosus* ScaCBA pili and *Streptococcus pneumoniae* RrgA pili have been described to be required for the phagocytic uptake by macrophages (Baorto et al., 1997; Wang et al., 2007; Orrskog et al., 2012; Vargas García et al., 2015). The absence of MrkA, however, did not affect macrophage phagocytosis, indicating that T3P are not required for this phenomenon. LPS and outer membrane proteins (OMPs) of *K. pneumoniae* have been reported to be involved in resistance to phagocytosis (March et al., 2013). Our group currently studies the effect of H-NS on other *K. pneumoniae* virulence factors such as LPS, OMPs, and siderophores.

Change of temperature is an environmental condition that affects the oligomerization state of H-NS and subsequently its DNA-binding properties, being crucial for control of transcriptional regulation (Ono et al., 2005; Stella et al., 2005, 2006). In bacteria such as *E. coli, Shigella* and *Salmonella*, virulence genes are thermoregulated by H-NS: down-regulated at low temperature and expressed at mammalian body temperature (37°C) (Maurelli and Sansonetti, 1988; Falconi et al., 1998; Umanski et al., 2002; Ono et al., 2005; Duong et al., 2007). In agreement with the thermoregulatory functions of H-NS on various virulence factors, fimbrial and capsular genes of *K. pneumoniae* were differentially repressed at 37 and 25°C, showing that H-NS-mediated repression is higher at the lower temperatures likely encountered outside of the host.

In summary, we have described for the first time the important role of H-NS in *K. pneumoniae*. Our data show that similar to other pathogens, *K. pneumoniae* H-NS is a master regulator assuring optimal expression of both T3P and CPS whose uncontrolled expression may severely impact bacterial fitness.

## Author contributions

Conceived and designed the experiments: MA, JF RR, MJ, MA, MD. Performed the experiments: MA, JF, RR, MJ MD. Analyzed the data: MA, JT, JG, MA, MD. Wrote the paper: MA, KV, MA, MD.

### Conflict of interest statement

The authors declare that the research was conducted in the absence of any commercial or financial relationships that could be construed as a potential conflict of interest.
